# Reduction of foot-and-mouth disease virus transmission in cattle vaccinated one or two weeks before challenge using a commercial polyvalent vaccine

**DOI:** 10.1016/j.jvacx.2020.100063

**Published:** 2020-04-13

**Authors:** Sergio Duffy, Norberto Fondevila, Sabrina Galdo Novo, María Aznar, Carlos Garro, Eliana Smitsaart, Gustavo Monti

**Affiliations:** a Instituto de Patobiología, CICVyA-INTA Castelar, Hurlingham 1686, Argentina; b Instituto de Virología, CICVyA-INTA Castelar, Hurlingham 1686, Argentina; c Servicio Nacional de Sanidad y Calidad Agroalimentaria (SENASA), Talcahuano 1660, Martínez 1640, Prov. Buenos Aires, Argentina; d Biogénesis Bagó S.A., Ruta Panamericana km 38.5, Garin 1619, Prov. Buenos Aires, Argentina; e Facultad de Ciencias Veterinarias, Universidad Austral de Chile, Independencia 641, Valdivia, Chile

**Keywords:** Foot-and-mouth disease, Vaccination, Cattle, Virus transmission

## Abstract

Immediate vaccination of the most susceptible and epidemiological relevant animals is a crucial part of control measures that facilitate virus elimination in case of entry of foot-and-mouth disease (FMD). The objective of this study was to evaluate the effect of cattle vaccination 7 and 14 days prior challenge using a vaccine commonly applied in systematic vaccination campaigns against transmission of FMD virus (FMDV). Transmission of FMDV was investigated in three groups of ten cattle each: one non-vaccinated group and two groups that were either vaccinated 7 days (−7/vaccinated group) or 14 days (−14/vaccinated group) before intranasal (IN) inoculation. Five cattle heads from each group were inoculated using the IN-route with the A/Argentina/2001 FMDV strain, while the remaining five cattle heads of each group were contact-exposed to inoculated cattle. Clinical signs were recorded; virus isolation and genome detection by RT-PCR were carried out on oesophageal–pharyngeal fluid (OPF) and blood. Neutralizing antibody titers and antibodies against non-structural proteins (NSP) of FMDV were also determined. Results suggest that the experimental design, virus challenge dose, and virus infectivity were appropriate and that the virus had been transmitted to naïve calves. Under the outlined experimental conditions, vaccination 7 and 14 days prior to challenge induced full clinical protection against virus inoculation. Moreover, −7/ or −14/vaccinated calves that had been contact-exposed to −7/ or −14/vaccinated IN-challenged calves, did not become infected. Consequently, no virus transmission occurred from vaccinated and subsequently infected calves to cohabitating vaccinated calves (R = 0). According to our results, early vaccination during an outbreak is effective as virus transmission can be significantly reduced using a vaccine commercially available, routinely applied in systematic vaccination campaigns.

## 1 Introduction

Foot-and-mouth disease (FMD) is a highly contagious disease of cloven-hoofed animals and considered the socioeconomic most important disease of livestock. The maintenance of the FMD-free status is a challenging task for countries free of FMD without and with vaccination. Introduction of FMDV into several FMD-free countries where vaccination is not practised caused severe epidemics (United Kingdom 2001, South Korea 2000–2002, among others). However, also FMD-free countries where vaccination is practised, such as Argentina (2006), Brazil (2005), Paraguay (2011), Colombia (2009–2017–2018), Republic of Korea (2010, 2014) and others, have experienced the introduction of FMDV [1]. Thus, measures to avoid introduction of FMDV and exposure of susceptible animals as well as establishment of early detection systems and contingency plans are needed to control and prevent outbreak of FMD.

The last FMD outbreak in Argentina was recorded in 2006 [1] and currently the whole country holds the FMD-free status. Argentina and other South American countries achieved FMD eradication through control strategies essentially based on systematic and mandatory vaccination of cattle. Although the overall cattle population has a high level of population immunity, highly susceptible groups of animals do exist such as calves with failure of passive immune transfer and/or a not yet developed immunity (i.e. calves that have not yet been vaccinated, or those that showed a poor response to vaccination, etc.).

Immediate vaccination of most susceptible and epidemiologically relevant animals is a decisive part of control measures to eliminate the virus in case of FMD-re-entry, if “vaccination to live” policy is followed. The effect of vaccination on an animal involves three aspects: (i) induction of protection against the disease (individual immunity), (ii) reduction of susceptibility of an individual animal (reduced infection risk), and (iii) reduction of infectivity or horizontal transmission within a herd (herd immunity) [2], [3]. It is of particular importance to understand the effect of emergency vaccination on the reduction of virus transmission.

The transmission of FMDV between susceptible species at a small-scale such as experimental studies and at higher-scales such as between farms, countries and regions has recently been reviewed [4], [5]. The current contingency plan of Argentina foresees that in case of an FMD-outbreak through reintroduction of strains against which the current vaccine strains are effective, a commercially available polyvalent immunogen will be applied. Accordingly, in an event of disseminated outbreak, FMD-free countries in which vaccination is not practiced may consider to acquire an important volume of doses from countries that have stocks of ready-to-use vaccines [6].

The effectiveness of a vaccine to prevent virus transmission within a vaccinated population is determined best in homogeneous groups. This implies that all animals in a group should either all be vaccinated or all be unvaccinated [2], [7]. Accordingly, the objective of this study was to evaluate the effect of cattle vaccination 7 and 14 days prior challenge using a commercial vaccine on the transmission of FMDV. In this study, we set out to test the suitability of a commercially available polyvalent vaccine, routinely used for systematic vaccination campaigns, to reduce virus transmission between vaccinated cattle.

## 2 Materials and methods

### 2.1 Animals and experimental design

Thirty naïve Holando-Argentino breed male calves with an average age of 216 days (range 210–225) and an average weight of 205 kg, were used. The experimental protocol followed biosecurity and animal welfare internal and federal regulations and was approved by the Institutional Committee for the Use and Care of Experimental Animals (CICUAE, CICVyA-INTA). Sample size of experimental groups were estimated following a previous report [8].

Fifteen days before challenge, animals were randomly allocated into three groups following a simple randomization procedure. Calves were vaccinated at the breeding farm 14 and 7 days before challenge and assigned to the −14/vaccinated (n = 10) and the −7/vaccinated (n = 10) group, respectively. Ten calves remained non-vaccinated (non-vaccinated group). Animals were transported to biocontainment animal facility 4 days before challenge.

One day before challenge, five calves of each group were assigned to be exposed by either intranasal or contact route through a simple randomization process. At the day of challenge, the five animals to be exposed by contact route from the −14/vaccinated, −7/vaccinated and non-vaccinated group were removed from their pens and housed in three other separate rooms. The five animals of each group that remained in the pen were challenged via the intranasal (IN) route.

Twenty-four hours after challenge, the initially separated animals were reunited with their original roommates and contact-exposed to IN-inoculated calves until the end of the study at 28 days post inoculation (dpi). The duration of the experiment was long enough for all susceptible animals to become infected and of those infected to recover before the end of the study.

### 2.2 Vaccine and vaccination

A commercial vaccine (Bioaftogen®, batch #688, Biogénesis Bagó, Argentina) consisted in a water-in-oil single emulsion containing the four FMDV strains O1 Campos, A24 Cruzeiro, A/Argentina/2001, and C3 Indaial with an antigen concentration greater than 30 µg per dose. The vaccine batch was examined by the Argentina National Food Safety and Quality Service (SENASA) and complied with requirements for safety, purity, and potency (in cattle) before its release to the market [9], [10]. The vaccine batch has been approved with an expected percentage of protection (EPP) of 96.3%, 95.7%, 98.0%, and 98.4% for O1 Campos, A24 Cruzeiro, A/Argentina/2001, and C3 Indaial vaccine strains, respectively, and has been applied in Argentine vaccination campaigns. Vaccination was performed using a single dose of 2 ml via the intramuscular route into the neck with 15-gauge, 18-mm needles on 10 ml disposable syringes.

In Argentina, this vaccine is routinely administered following local vaccination programme guidelines. The vaccination frequency is twice a year in cattle younger than 2 years old and once a year in those older than 2 years.

### 2.3 Virus challenge

Before challenge, calves were sedated using xylazine, 0.22 mg/kg, via the intramuscular route. Challenge was performed via the IN-route, inoculating 10,000 TCID50% of A/Argentina/2001 FMDV strain per calf, in a volume of 1 ml per nostril. The A/Argentina/2001 viral strain belongs to SENASA reference collection. As inoculum the second cattle passage of a field isolate from Trenque Lauquen, Buenos Aires province, Argentina obtained during the 2001 outbreak was used [11]. The day of challenge was defined as day 0 (0 dpi).

### 2.4 Clinical inspection and sampling

Clinical signs and rectal temperature were recorded daily from 0 to 14 dpi and later at 14, 21 and 28 dpi. Oesophageal-pharyngeal fluid (OPF) and EDTA-anticoagulated blood samples were collected at 0, 1, 3, 5, 7, 9, 11, 14, 21 and 28 dpi. Clotted blood for serology was collected at −14, −7, 0, 7, 9, 14, and 28 dpi from animals of all groups. Sera were separated and stored at −20 °C until further use. OPF sampling was carried out following the probang technique [12].

### 2.5 Virus isolation

Monolayers of BHK-21 in 96-well plates were used. OPF and anticoagulated blood samples were inoculated in quadruplicate, performing three passages of 48 h each, and examined for cytopathic effect. The positive samples were titrated in tenfold serial dilutions from undiluted to 10^-6^ dilution. The virus titers calculated by the Reed and Muench Method [13] was expressed as TCID50%/ml.

### 2.6 RNA extraction and real time RT-PCR (RT-PCR)

The procedures for RNA extraction and real time RT-PCR were followed with minor modifications as described in the OIE terrestrial manual [14]. Two hundred and fifty ml of either OPF or of an anticoagulated blood sample were mixed with 1000 µl Trizol® and RNA was extracted following the instruction of the manufacturer. Viral RNA in samples was reverse transcribed using random hexamers and then quantified by real-time RT-PCR using primers targeting the 3D polymerase region [15]. The reaction was performed in a Roche Light Cycler 2.0 thermocycler as previously described [16]. Samples that presented a geometric increase in fluorescence emission in two successive cycles prior to cycle number 40 were scored positive, and the first of the two cycles showing emission elevation was considered to be the first cycle of positivity (CP). To convert cycle threshold values generated by real time RT-PCR from experimental samples to RNA genome copies per milligram, serial 10-fold dilutions of plasmid I38 containing the 3D sequence of FMDV (kindly provided by Dr. Soledad Nuñez, Institute of Biotechnology, INTA-Castelar) were analyzed as a quantitative positive control. The number of moles of RNA were calculated as = CP × -0.25 + 11.68.

### 2.7 Serology

Virus neutralization test (VNT) was carried out as described previously [14], using BHK-21 cell suspensions and A/Argentina/2001 virus.

Pre- and post-challenge serum samples were tested for the presence of antibodies directed against the non-structural 3ABC polypeptide of FMDV using two commercially available ELISA kits, the PrioCHECK FMDV NS test [Prionics Lelystad, The Netherlands] and the Screening Test NCPanaftosa-Bovine [PANAFTOSA, PAHO/WHO, Rio de Janeiro, Brazil]. Additionally, serum samples collected at 28 dpi were determined by the NCPanaftosa Confirmatory Test-Bovine, which consisted in an enzyme-linked immunoelectrotransfer blot [EITB] assay. Assays were performed as per manufacturer’s instructions.

### 2.8 Statistical analysis

We analyzed four indicators of infection in each group, and applied statistical analysis to compare them [17]. The duration of OPF virus excretion was estimated as the number of days from the first to the last day the virus could be isolated from each animal until day 14 post infection. The mean daily virus detection (MDVD) in OPF was estimated as the average of TCID50%/ml per day in each group for the days the virus was detected. The duration of viral genome detection in OPF was estimated as the number of days from the first to the last day the viral genome was detected by RT-PCR until day 14 post infection. The mean daily viral genome detection (MDVGD) in OPF was estimated as the average per day for the days viral RNA was detected by RT-PCR.

Kruskal-Wallis test was used to analyze differences between the three groups. When differences were statistically significant, pairwise comparison between groups were performed (Statistix 8.0©). When a comparison between two groups were required, Wilcoxon rank sum test was applied (Statistix 8.0©). A stochastic susceptible-infectious-removed (SIR) model was used to estimate the reproduction ratio R by maximum likelihood [8]. The estimator of R was based on the final size of the outbreak observed in the experiments. R is defined as the average number of new cases of an infection. Comparison between R in non-vaccinated and vaccinated calves was done by testing that R is higher in vaccinated than in non-vaccinated calves (one-tailed test) [8].

## 3 Results

### 3.1 Clinical signs

All non-vaccinated calves that were IN-challenged or contact-exposed developed generalized FMD (Table 1). In the group of IN-challenged non-vaccinated animals, lesions compatible with FMD were detected in the interdigital space, coronary band, tongue, gums, and nostrils. All animals in this group showed severe lesions in two or more of the above mentioned sites and fever (>40 °C) between 3 and 7 dpi. One of the 5 IN-challenged calves evidenced lesions after 3 dpi and the remaining animals after 5 dpi (data not shown).

**Table 1:** Virus isolation from oesofagueal-pharyngeal fluid (OPF) samples and blood, detection of viral RNA in OPF, clinical signs and antibodies to non-structural proteins (NSP).

1 – N = no clinical signs; clinical score was determined by the number of feet presenting FMD lesions plus the presence of lesions in the tongue, gum and nostrils; the maximum score is 7. 2 – Detection of antibodies to NSP by PrioCHECK FMDV NS and Screening Test NCPanaftosa-Bovine.

NEG: non-reactive; the first number indicates the last day in which the sample was non-reactive and, the second number indicates the first day in which the sample was reactive.

3 – TCID 50%/ml of FMDV detected in OPF test; * days after infection (dpi) in which virus was detected in blood; ** TCID 50%/ml of FMDV detected in blood.

Cells in gray indicate viral RNA detection by rt RT-PCR in OPF; for quantitative results refer to Table 2; † died at 11 dpi and has been non-reactive to NSP antibodies until 11 dpi.

In the group of contact-exposed non-vaccinated calves, two animals presented lesions from 5 dpi, and three from 7 dpi on (data not shown), and fever was detected between 5 and 9 dpi. One calf died 11 dpi due to causes not related to FMD. In contrast, neither IN-challenged nor contact-exposed vaccinated animals developed clinical signs after challenge.

### 3.2 Virus isolation

Virus in OPF was isolated from all IN-challenged calves with the exception of one calf of the −7/vaccinated group (#715) (Table 1). In contrast, among contact-exposed calves, virus was isolated only from animals belonging to the non-vaccinated group (Table 1, Table 3). Regarding the number of days of virus detection (OPF), pairwise comparisons among groups showed significant differences exclusively between the IN-challenged non-vaccinated and the −7/vaccinated groups (P < 0.05). In addition, the MDVD was significantly different only between the IN-challenged non-vaccinated group (TCID50%/ml = 4.25) and the −7/vaccinated group (TCID50%/ml = 1.95) (P < 0.05; Table 3).

Virus was isolated from blood from four IN-challenged animals and from four animals of the contact-exposed non-vaccinated calves (Table 1). In vaccinated groups, viremia was detected in a single calf (#729) belonging to the −14/vaccinated group subjected to IN inoculation at 3 and 5 dpi.

Regarding the duration of virus detection and the MDVD in OPF in unvaccinated calves, no significant differences (P > 0.05) were found among calves exposed by contact to those IN-challenged (Table 3).

### 3.3 Real time RT-PCR

Viral genome was detected in OPF of all IN-challenged calves and in the contact-exposed non-vaccinated calves, yet not in OPF of contact-exposed calves that had been vaccinated (Table 2). No significant differences were observed in the duration of viral genome detection in OPF among the IN-challenged −7/vaccinated, −14/vaccinated, and non-vaccinated group (P > 0.05; Table 3). Regarding MDVGD in OPF, pairwise comparisons among groups showed significant differences only between the IN-challenged non-vaccinated (5.51) and the −7/vaccinated group (3.27) (P < 0.05; Table 3). Regarding the duration of viral genome detection and MDVGD in OPF in unvaccinated calves, no significant differences (P > 0.05) were found between contact-exposed and IN-challenged calf groups (Table 3).

**Table 2:** Detection of viral RNA in oesofagueal-pharyngeal fluid samples and blood.

			Days after challenge																			
			0		1		3		5		7		9		11		14		21		28	
Animal Group	Calf #	Viral exposure	Blood	OPF	Blood	OPF	Blood	OPF	Blood	OPF	Blood	OPF	Blood	OPF	Blood	OPF	Blood	OPF	Blood	OPF	Blood	OPF
Vaccinated −7	720	Contact	–	–	–	–	–	–	–	–	–	–	–	–	–	–	–	–	–	–	–	–
	723	Contact	–	–	–	–	–	–	–	–	–	–	–	–	–	–	–	–	–	–	–	–
	730	Contact	–	–	–	–	–	–	–	–	–	–	–	–	–	–	–	–	–	–	–	–
	736	Contact	–	–	–	–	–	–	–	–	–	–	–	–	–	–	–	–	–	–	–	–
	737	Contact	–	–	–	–	–	–	–	–	–	–	–	–	–	–	–	–	–	–	–	–
	704	Intranasal	–	–	–	4.77*	–	2.28	–	3.01	–	2.77	–	1.72	–	1.11	–	1.81	–	–	–	–
	715	Intranasal	–	–	–	1.11	–	1.11	–	–	–	–	–	–	–	–	–	–	–	–	–	–
	724	Intranasal		–	–	2.7		–	–	1.76	–	–	–	1.91	–	1.11	–	1.11	–	–	–	1.11
	727	Intranasal	–	–	–	1.11	–	1.66	–	2.60	–	2.71	–	2.55	–	2.38	–	1.11	–	–	–	1.11
	728	Intranasal	–	–	–	2.45	–	1.11	–	2.70	–	2.6	–	2.46	–	2.49	–	1.11	–	1.11	–	1.11

Vaccinated −14	696	Contact	–	–	–	–	–	–	–	–	–	–	–	–	–	–	–	–	–	–	–	–
	714	Contact	–	–	–	–	–	–	–	–	–	–	–	–	–	–	–	–	–	–	–	–
	719	Contact	–	–	–	–	–	–	–	–	–	–	–	–	–	–	–	–	–	–	–	–
	726	Contact	–	–	–	–	–	–	–	–	–	–	–	–	–	–	–	–	–	–	–	–
	731	Contact	–	–	–	–	–	–	–	–	–	–	–	–	–	–	–	–	–	–	–	–
	694	Intranasal	–	–	–	2.60	–	3.19	–	4.02	–	1.11	–	2.62	–	3.13	–	–	–	1.67	–	1.11
	705	Intranasal	–	–	–	2.81	–	3.27	–	3.60	–	1.11	–	–	–	1.11	–	–	–	1.11	–	–
	729	Intranasal	–	–	–	2.99	2.82	3.13	–	4.14	–	2.73	–	1.99	–	1.11	–	1.11	–	–	–	–
	732	Intranasal	–	–	–	3.82	–	2.91	–	2.06	–	2.97	–	1.11	–	2.43	–	1.11	–	–	–	1.67
	734	Intranasal	–	–	–	3.35	–	3.12	–	1.99	–	2.21	–	2.26	–	2.25	–	2.40	–	1.11	–	–

No vaccinated	699	Contact	–	–	–		–	1.11	–	5.69	3.37	5.77	1.11	5.3	–	5.31	–	2.53	–	–	–	2.17
	701	Contact	–	–	–	–	–	–	–	4.36	–	4.93	–	3.69	–	1.99	–	–	–	2.57	–	–
	702	Contact	–	–	–	–	–	–	2.24	5.82	4.49	4.47	–	4.33	1.11	3.47	–	–	–	–	–	–
	707	Contact	–	–	–		–	2.13	–	4.39	2.96	3.64	–	3.67	–	2.41	–	–	–	–	–	–
	712	Contact	–	–	–	–	–	1.11	–	5.70	3.43	4.64	–	4.08	1.11	2.95	†	†	†	†	†	†
	693	Intranasal	–	–	–	4.07	3.23	4.92	3.94	6.27	–	4.08	–	2.50	–	3.39	–	–	–	–	–	–
	703	Intranasal	–	–	–	2.87	–	4.27	3.94	6.47	1.11	4.45	–	3.19	–	2.76	–	–	–	–	–	–
	706	Intranasal	–	–	–	4.14	–	3.01	–	6.65	–	4.44	–	3.37	–	1.11	–	–	–	–	–	1.11
	735	Intranasal	–	–	–	2.88	2.83	3.39	3.95	5.07	–	4.27	–	2.09	–		–	–	–	1.11	–	–
	738	Intranasal	–	–	–	1.77	2.33	4.05	3.88	4.69	–	3.51	–	2.50	–	2.73	–	–	–	–	–	–

* log10 copies of viral RNA/ml; - no genome detected; † died at 11 dpi due to causes not related to FMD.

**Table 3:** Infection parameters and statistics^1^ applied for comparisons among experimental groups.

	Intranasal			Contact		
Parameters (range 1–14 days post infection)	−14/ vacc	−7/ vacc	Non-vaccinated	−14/ vacc	−7/ vacc	Non-vaccinated
Duration of virus excretion in OPF (days)	9.2^ab^	13.3^a^	7.0^b^	NVD	NVD	6.8
Mean daily virus detection (MDVD) in OPF	2.90^ab^	1.95^a^	4.25^b^	NVD	NVD	4.06
Duration of viral genome detection in OPF	12.80^a^	12.20^a^	11.00^a^	NGD	NGD	9.2
Mean daily viral genome detection (MDVGD)^2^ in OPF (days)	3.20^ab^	3.27^a^	5.51^b^	NGD	NGD	5.16

NVD: no virus detected; NGD: no genome detected; different letters mean significant differences between groups challenged intranasally (P ≤ 0.05); there were no significant differences in any indicator between animals challenged intranasally and by contact in the non-vaccinated group (P > 0.05).

1 Kruskal-Wallis test. Wilcoxon Rank sum test (comparison between two groups).

2 log10 copies of viral RNA/ml.

In blood samples, viral genome could be detected from 4 out of 5 IN-challenged calves and contact-exposed calves of the non-vaccinated groups (Table 2). Importantly, this result corresponds with the detection of virus in blood sample in 4 out of 5 calves in each non-vaccinated group (Table 1). Furthermore, as in the detection of viremia, the viral genome was detected with delay of one sampling in the in contact-exposed calves regarding the IN-challenged calves. In the non-vaccinated calves, viral genome in blood samples was detected until 7 dpi in the IN-challenged group and until 11 dpi in the contact-exposed calf group.

Viral genome in blood was not detected in contact-exposed vaccinated animals. (Table 2). Regarding the IN-challenged vaccinated calves no viral genome in blood was detected in the −7/vaccinated group. However, 1 out of 5 animals presented viral genome in the −14/vaccinated group, consistent with virus isolation in blood on 3 and 5 dpi (#729, Table 1).

### 3.4 Serology

**Fig. 1:**
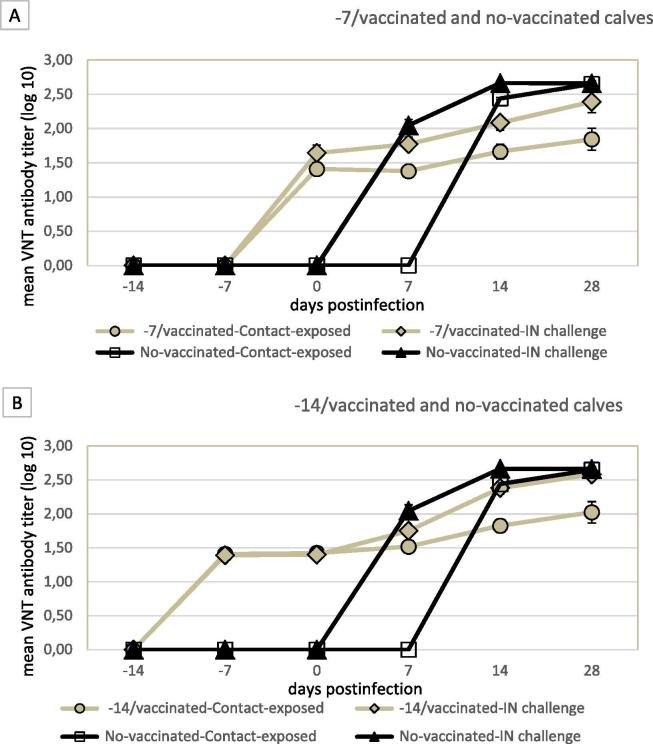
Mean virus neutralization test (VNT) antibody titers against A/Argentina/2001 of −7/vaccinated group (A) and −14/vaccinated group (B) in comparison with no-vaccinated group. Vertical lines indicate the standard error of the mean (SEM). No significant differences were detected among groups (P > 0.05).

Vaccination induced in calves neutralizing antibodies to A/Argentina/2001 FMDV as early as 7 dpv (Fig. 1). The vaccinated and IN-challenged calves had a higher mean titer than vaccinated and contact exposed calves from 7 dpi on at each bleeding time, although observed differences were no significant (P > 0.05). The kinetics of neutralizing antibodies of each group is shown in Fig. 1.

Regarding seroconversion to NSP antibodies, all IN-inoculated cattle (with the exception of animal #715 of the −7/vaccinated group) developed NSP antibodies. In non-vaccinated IN-challenged and contact-exposed groups, NSP antibodies were detected from 9 and 14 dpi, respectively, whereas in vaccinated IN-challenged groups, NSP antibodies were detected from 14 dpi. Vaccinated inoculated calves that scored positive showed T/C values between 1 and 2 by NCPanaftosa-Bovine (cut-off value T/C ≥ 1) and PI values between 51 and 69% by PrioCHECK FMD NS (cut-off value PI ≥ 50%), whereas non-vaccinated inoculated calves showed T/C values above 2 by NCPanaftosa-Bovine and PI values above 70% by PrioCHECK FMD NS (data not shown). Noteworthy, of non-vaccinated calves challenged by contact, one calf died at day 11 dpi due to an undetermined cause not related to FMD. None of the contact exposed −14 and −7/vaccinated groups developed detectable antibodies to NSP at any time during the study (Table 1). Results obtained by both ELISA kits were equivalent, with the exception of animal #727 of the IN-challenge −7/vaccinated group that showed reactivity to 3ABC only by PrioCHECK FMDV NS test at 28 dpi (PI value = 51%) and not by the other method.

### 3.5 Viral transmission

All IN-challenged calves and all non-vaccinated calves that had been contact-exposed became infected. Conversely, none of the contact-exposed animals of-14/ and-7/vaccinated groups became infected.

The R_NoVacc_ was estimated as∞ (95% CI, 0.67-∞), which is not significantly > 1 (P = 0.083). For both groups, R_Vacc-14_ and R_Vacc-7_ was estimated to be 0 (95% CI, 0–2.18), which is not significantly < 1 (P = 0.13). Comparison between R_NoVacc_ and either R_Vacc-14_ or R_Vacc-7_ showed a significant difference (P = 0.013).

## 4 Discussion

The knowledge of the effect of vaccination to prevent virus transmission is crucial for the design of control measures including emergency vaccination and prediction of virus dissemination in an event of an outbreak. Previous studies in cattle determined the effectiveness of vaccination in inducing early protection [18], [19]. Correspondingly, in our study, calves vaccinated either 7 or 14 days prior to IN-inoculation were protected against clinical disease. Previous experiments also showed the capacity of vaccination in reducing virus transmission in cattle [5].

The aim of our study was to provide additional data on FMDV transmission among non-vaccinated and vaccinated calves using a regular commercially available vaccine applied in vaccination campaigns.

As expected, IN-inoculation of non-vaccinated calves with FMDV induced clinical disease, shedding of FMDV, viremia, seroconversion to NSP, and generation of neutralizing antibodies indicating successful inoculation. During the FMD outbreak in Argentina in 2001, the A/Argentina/2001 strain showed a high transmissibility within and between herds, and was therefore used in this study for challenge. As infection parameters demonstrated, IN-inoculated calves transmitted the virus to cohabitating naïve calves (R = ∞).

Duration of genome excretion in OPF was similar between IN-challenged groups of non-vaccinated, −7/, and −14/vaccinated calves, demonstrating the very high virus challenge doses applied in our experiment. In comparison to virus isolation, duration of genome detection in non-vaccinated animals was longer in the IN-challenged than in the contact-exposed group, which may be explained by the higher sensitivity of the RT-PCR assay as compared to virus isolation. In overall, these data demonstrate that the experimental design, challenge virus dose and virus infectivity were appropriate and that virus transmission from naïve IN-challenged calves to naïve calves took place.

One animal of the −7/vaccinated IN-challenged group (#728) showed at 28 dpi virus detection in OPF and became “carrier”. This situation is not unexpected as vaccination protects against clinical signs, and a proportion of animals may remain subclinically infected and turn into carriers [20].

The detection of viremia and viral genome in a single vaccinated calf (#729, from the −14/vaccinated IN challenged group) has been suggestive for low virus replication that did not lead to dissemination and development of vesicles in epithelial areas. This finding may be explained by the fact that the animal #729 showed the lowest neutralizing titer of this group on the day of challenge (data not shown). Previous studies did not find virus in the blood of vaccinated inoculated cattle, even in the presence of virus or virus genome in OPF samples [7], [18], [21]. This difference with regard to our finding may be due to challenge methods, dose of challenge, virulence of the strain of challenge, vaccine potency, among others.

Regarding seroconversion to NSP, it should be pointed out that antibodies to NSP were detected later in IN-challenged vaccinated groups (from 14 dpi) than in the IN-challenged non-vaccinated group (from 9 dpi), along with lower ELISA values, probably due to the effect of vaccination that limit viral replication [22]. Additionally, no NSP antibodies were detected in animal #727 from −7/vaccinated challenged group by NCPanaftosa-Bovine, but it was found positive by PrioCHECK FMDV NS at 28 dpi. This late detection of NSP antibodies in cattle in which virus was isolated from OPF was not unexpected. The discrepancy in the NSP antibody detection between both tests may be explained by the borderline result of this serum (PI value = 51%) in PrioCHECK FMDV NS that resulted in a nonreactor by NCPanaftosa-Bovine test, frequent finding in samples near the detection limit as the diagnostic sensitivity reported by both tests was similar [22].

Under the applied experimental conditions, vaccination 7 or 14 days prior to challenge induced full clinical protection against IN virus inoculation. Moreover, −7/ or −14/vaccinated calves that had been contact-exposed to-7/ or −14/vaccinated IN-inoculated calves, did not become infected. Consequently, no virus transmission occurred from vaccinated and subsequently infected calves to cohabitating vaccinated calves (R = 0).

The registered vaccine in Argentina is composed of 4 vaccine strains, including C3 Indaial, as well as the vaccine authorized in USA and Canada for emergency situation [6], [23]. Although there were no reports of serotype C in the world since 2004, the authorized vaccine in Argentina requires the inclusion of serotype C since 2006 [24]. Regarding potency, the vaccines manufactured in Argentina are formulated to meet a minimum of 75% of EPP (or minimum of 3 PD50). However, most of the batches released to the market showed >85% of EPP in the potency test in cattle conducted by SENASA [25]. Additionally, a commercial vaccine similar to the one used in our study has shown early immunity in cattle [26]. Unlike previous studies in cattle in which high-potency vaccines were used [7], [21], [27], this study used a polyvalent vaccine that is regularly administered in systematic vaccination campaigns in Argentina. This has the added advantage that in case of an epidemic outbreak is faced, the vaccine would be rapidly available in large quantities. In this sense, rolling stocks of ready-to-use polyvalent vaccines have been considered as a feasible vaccine reserve to respond rapidly to outbreaks [28]. Mostly, FMD free countries have Antigen Banks through contracts with vaccine manufacturing companies. In the case of disseminated outbreaks in high density areas, the number of doses from Antigen Banks may be insufficient, and therefore the use of ready-to-use vaccines from countries that apply such vaccines in their vaccination programs has been proposed [29]. In agreement with the results presented in this controlled study, field evidence on the effects of vaccination reducing within herd transmission was reported by Brito et al. [30] in which the protective effect of the vaccine was evidenced by the association between vaccination and low rate of within herd transmission.

The full protection and the lack of virus transmission between vaccinated calves at an early stage after vaccination, as observed under the outlined experimental conditions, are key features for effective contingency policies. According to our results, vaccination seems to be a suitable measure to control an outbreak because it has the potential to reduce significantly virus transmission under the premise that the corresponding vaccine is rapidly applied.

## 5 Conclusions

We studied the effect of vaccination on the transmission of FMDV in cattle using a commercially available polyvalent vaccine. Our work demonstrated that vaccination 7 and 14 days prior to challenge induced full clinical protection against virus inoculation. Additionally, the vaccinated calves that had been contact-exposed to vaccinated and inoculated calves did not become infected. Thus, no virus transmission occurred from vaccinated and subsequently infected calves to cohabitating vaccinated calves.

According to our results, early vaccination during an outbreak is effective as virus transmission can be significantly reduced using a commercial polyvalent vaccine, routinely used in systematic vaccination campaigns.

## CRediT authorship contribution statement

**Sergio Duffy:** Conceptualization, Formal analysis, Funding acquisition, Supervision, Writing - original draft. **Norberto Fondevila:** Investigation, Methodology. **Sabrina Galdo Novo:** Investigation, Methodology. **María Aznar:** Data curation, Formal analysis. **Carlos Garro:** Data curation, Formal analysis. **Eliana Smitsaart:** Visualization, Writing - review & editing. **Gustavo Monti:** Conceptualization.

## Declaration of Competing Interest

The authors declare the following financial interests/personal relationships which may be considered as potential competing interests: Smitsaart E, employee of Biogenesis Bago, declares that its judgment and objectivity was not biased by their contractual condition. Bioaftogen® is a registered trademark of Biogenesis Bago.

## Appendix A Supplementary material

Supplementary data 1 Description of animal facility and movement of the personnel involved in handling of animals during the experiment.

## References

[1] OIE. World Animal Health Information Database (WAHIS) Interface Version 1. Animal Health in the World; 2018.

[2] De Jong M.C., Bouma A. (2001). Herd immunity after vaccination: how to quantify it and how to use it to halt disease. Vaccine.

[3] De Jong M.C., Kimman T.G. (1994). Experimental quantification of vaccine-induced reduction in virus transmission. Vaccine.

[4] Paton D.J., Gubbins S., King D.P. (2018). Understanding the transmission of foot-and-mouth disease virus at different scales. Curr Opin Virol.

[5] Orsel K., Bouma A. (2009). The effect of foot-and-mouth disease (FMD) vaccination on virus transmission and the significance for the field. Can Vet J.

[6] Roth JA, Spickler AR. FMD Vaccine Surge Capacity for Emergency Use in the United States. Center for Food Security and Public Health at Iowa State University; 2014. Available at: http://www.cfsph.iastate.edu/pdf/fmd-vaccine-surge-capacity-for-emergency-use-in-the-US.

[7] Orsel K., Dekker A., Bouma A., Stegeman J.A., de Jong M.C. (2005). Vaccination against foot and mouth disease reduces virus transmission in groups of calves. Vaccine.

[8] Kroese AH, De Jong MCM. Design and analysis of transmission experiments. Society for Veterinary Epidemiology and Preventive Medicine, Proceedings, Noordwijkerhout, The Netherlands; 2001. pp. xxi-xxxvii.

[9] SENASA. Act No. 351/2006. In: Boletín Oficial No. 30.940, Argentina, July 5th 2006; 2006. Available from: http://servicios.infoleg.gob.ar/infolegInternet/verNorma.do?id=117636.

[10] SENASA. Act No. 111/20In: Boletin Oficial No 31855, Argentina; 2010. Available at: http://servicios.infoleg.gob.ar/infolegInternet/verNorma.do?id=164766.

[11] Mattion N., Konig G., Seki C., Smitsaart E., Maradei E., Robiolo B. (2004). Reintroduction of foot-and-mouth disease in Argentina: characterisation of the isolates and development of tools for the control and eradication of the disease. Vaccine.

[12] Sutmoller P., Cottral G.E. (1967). Improved techniques for the detection of foot-and-mouth disease virus in carrier cattle. Arch Gesamte Virusforsch.

[13] Reed L.J., Muench H. (1938). A simple method of estimating fifty per cent endpoints. J Hygiene.

[14] OIE. Manual of Diagnostic Tests and Vaccines for Terrestrial Animals. Foot and mouth disease (infection with foot and mouth disease virus), World Organization for Animal Health, Paris, France; 2017.

[15] Callahan J.D., Brown F., Osorio F.A., Sur J.H., Kramer E., Long G.W. (2002). Use of a portable real-time reverse transcriptase-polymerase chain reaction assay for rapid detection of foot-and-mouth disease virus. J Am Vet Med Assoc.

[16] Fondevila N., Compaired D., Maradei E., Duffy S. (2014). Validation of a real time RT-PCR assay to detect foot-and-mouth disease virus and assessment of its performance in acute infection. Rev Argent Microbiol.

[17] Eble P.L., Bouma A., de Bruin M.G., van Hemert-Kluitenberg F., van Oirschot J.T., Dekker A. (2004). Vaccination of pigs two weeks before infection significantly reduces transmission of foot-and-mouth disease virus. Vaccine.

[18] Golde W.T., Pacheco J.M., Duque H., Doel T., Penfold B., Ferman G.S. (2005). Vaccination against foot-and-mouth disease virus confers complete clinical protection in 7 days and partial protection in 4 days: Use in emergency outbreak response. Vaccine.

[19] Cox S.J., Voyce C., Parida S., Reid S.M., Hamblin P.A., Paton D.J. (2005). Protection against direct-contact challenge following emergency FMD vaccination of cattle and the effect on virus excretion from the oropharynx. Vaccine.

[20] Doel T.R., Williams L., Barnett P.V. (1994). Emergency vaccination against foot-and-mouth disease: rate of development of immunity and its implications for the carrier state. Vaccine.

[21] Orsel K., de Jong M.C., Bouma A., Stegeman J.A., Dekker A. (2007). The effect of vaccination on foot and mouth disease virus transmission among dairy cows. Vaccine.

[22] Brocchi E., Bergmann I.E., Dekker A., Paton D.J., Sammin D.J., Greiner M. (2006). Comparative evaluation of six ELISAs for the detection of antibodies to the non-structural proteins of foot-and-mouth disease virus. Vaccine.

[23] CFIA. Veterinary biologics licensed by the Canadian Centre for Veterinary Biologics for commercial distribution and sale in Canada. Emergency preparedness; 2020.

[24] SENASA. Act No. E 609/2017 In: Boletín Oficial No.: 33714, Argentina; 2017. Available at: http://servicios.infoleg.gob.ar/infolegInternet/verNorma.do?id=279809.

[25] Smitsaart E., Espinoza A.M., Maradei E., Cosentino B., Guinzburg M., Madonni G. (2015). Importance of foot and mouth disease vaccine purity in interpreting serological surveys. Rev Sci Tech.

[26] Quattrocchi V., Pappalardo J.S., Langellotti C., Smitsaart E., Fondevila N., Zamorano P. (2014). Early protection against foot-and-mouth disease virus in cattle using an inactivated vaccine formulated with Montanide ESSAI IMS D 12802 VG PR adjuvant. Vaccine.

[27] Bravo de Rueda C., de Jong M.C., Eble P.L., Dekker A. (2015). Quantification of transmission of foot-and-mouth disease virus caused by an environment contaminated with secretions and excretions from infected calves. Vet Res.

[28] Lombard M., Fussel A.E. (2007). Antigen and vaccine banks: technical requirements and the role of the European antigen bank in emergency foot and mouth disease vaccination. Rev Sci Tech.

[29] Spickler AR, Roth JA. NAHEMS Guidelines: Vaccination for Contagious Diseases, Appendix A: Foot-and-Mouth Disease. Veterinary Microbiology and Preventive Medicine Reports. 2; 2015. Available at: http://lib.dr.iastate.edu/vmpm_reports/2.

[30] Brito B.P., Perez A.M., Cosentino B., Rodriguez L.L., Konig G.A. (2011). Factors associated with within-herd transmission of serotype A foot-and-mouth disease virus in cattle, during the 2001 outbreak in Argentina: a protective effect of vaccination. Transbound Emerg Dis.

