# Supplementary material for: Reduction of foot-and-mouth disease virus transmission in cattle vaccinated one or two weeks before challenge using a commercial polyvalent vaccine
Source: Vaccine X. 2020 Apr 13;5:100063. doi: 10.1016/j.jvacx.2020.100063 (PMC7190753; doi:10.1016/j.jvacx.2020.100063)
Supplement: Supplementary data 1 — Description of animal facility and movement of the personnel involved in handling of animals during the experiment. [file mmc1.docx]

**Supplementary File**

The biosecurity level 4 facility is composed of 6 independent pens, each one 5.6 m long x 5.5 m wide, and 3.6 m high. The net surface available for animals is 4.8 m x 5.5 m. Each pen includes 4 feeders that measure 1 m long x 0.5 m wide, and 0.5 m high and one drinking fountain of 0.5 m x 0.5 m x 0.5 m. These dimensions allowed frequent direct contact between animals housed in the same room. The air is introduced by an injection mouth and extracted through two mouths and 20 renewals of air per hour are carried out. Each animal group to be challenged was kept in independent pens within the biosecurity facility. No additional animal entered the building during the experiment.

The staff that handled non-vaccinated and challenged calves had no contact with animals of other groups during the experiment and was involved in the virus inoculation, animal movements, clinical inspection, sample collection, and cleaning of rooms. The same tasks were carried out on vaccinated groups before their challenge by other personnel. Every day these personnel started with the clinical inspection and sampling of the animals of the -14/vaccinated group, and continued then with the -7/vaccinated group.
